# Context-aware modeling of neuronal morphologies

**DOI:** 10.3389/fnana.2014.00092

**Published:** 2014-09-05

**Authors:** Benjamin Torben-Nielsen, Erik De Schutter

**Affiliations:** ^1^Computational Neuroscience Unit, Okinawa Institute of Science and Technology Graduate UniversityOnna son, Japan; ^2^Theoretical Neurobiology and Neuroengineering, University of AntwerpWilrijk, Belgium

**Keywords:** dendrite, morphology, computational modeling, growth cone, extracellular space

## Abstract

Neuronal morphologies are pivotal for brain functioning: physical overlap between dendrites and axons constrain the circuit topology, and the precise shape and composition of dendrites determine the integration of inputs to produce an output signal. At the same time, morphologies are highly diverse and variant. The variance, presumably, originates from neurons developing in a densely packed brain substrate where they interact (e.g., repulsion or attraction) with other actors in this substrate. However, when studying neurons their context is never part of the analysis and they are treated as if they existed in isolation. Here we argue that to fully understand neuronal morphology and its variance it is important to consider neurons in relation to each other and to other actors in the surrounding brain substrate, i.e., their context. We propose a context-aware computational framework, NeuroMaC, in which large numbers of neurons can be grown simultaneously according to growth rules expressed in terms of interactions between the developing neuron and the surrounding brain substrate. As a proof of principle, we demonstrate that by using NeuroMaC we can generate accurate virtual morphologies of distinct classes both in isolation and as part of neuronal forests. Accuracy is validated against population statistics of experimentally reconstructed morphologies. We show that context-aware generation of neurons can explain characteristics of variation. Indeed, plausible variation is an inherent property of the morphologies generated by context-aware rules. We speculate about the applicability of this framework to investigate morphologies and circuits, to classify healthy and pathological morphologies, and to generate large quantities of morphologies for large-scale modeling.

## INTRODUCTION

Neuronal morphology is important for brain functioning. The interplay between dendritic and axonal morphology limits the microcircuits (Peters and Payne, 1993), and the shape and composition of dendrites define how inputs are integrated to produce outputs (London and Häusser, 2005; Silver, 2010; Torben-Nielsen and Stiefel, 2010). As such, it is not surprising that changing morphological traits and morphological anomalies are implicated in neuro-developmental and degenerative diseases (Kaufmann and Moser, 2000; Dierssen and Ramakers, 2006). Nevertheless, neurons come in all shapes and sizes. The diversity is said to express the difference between neuron classes while variation represents the intra-class differences (Soltesz, 2005). Diversity originates from the genetic make-up of neurons (Jan and Jan, 2010; Tavosanis, 2012). By contrast, the variance can be assumed to originate from interactions between the developing neuron and the brain substrate, its context (McAllister, 2000; Scott and Luo, 2001; Landgraf and Evers, 2005; Jan and Jan, 2010; Tavosanis, 2012). Indeed, in both axonal (Mortimer et al., 2008) and dendritic (Gao, 2007; Cove et al., 2009) development a plethora of microscopic interactions have been revealed to influence branching patterns and “guide” the direction of growth. Thus, a neuron’s context holds the key to understanding morphological variance.

Unfortunately, the context surrounding a neuron has historically been neglected in the analysis and quantification of morphologies. In a highly influential work, Hillman argued that dendritic morphologies could be described completely and accurately by a finite set of morphometric descriptors (Hillman, 1979). Thus, the idea was born that careful description of morphometrics measured from isolated neurons would be sufficient to characterize neuronal morphology. Later, when digital reconstructions became more common practice, this idea inspired the way neurons are represented digitally: a pure representation of the morphology itself without any information about the context. Currently, a digital representation consists of a set of points in three dimensions with additional information on how they are linked to each other, as is done in the *de facto* standard SWC format (Cannon et al., 1998).

As a consequence, morphometric features used to quantify and analyze morphologies (such as the order and degree of points in the neuronal tree or neurite lengths) relate to the neuron itself and are unable to describe any characteristic of the context. Hence, statistical approaches to analyze morphologies and their variance that use these morphometric features are bound to fail to describe neuronal morphologies correctly as contextual influences including boundaries, capillaries and other neurons, cannot be taken into account. Indeed, in earlier work it was shown that the variance in morphometric features can be so high that no statistical model can be constructed to accurately describe the limited data (Torben-Nielsen et al., 2008).

An alternative, albeit in practice closely related to the pure statistical approach to study neuronal morphologies is the so-called “generative approach” (Ascoli et al., 2001; van Pelt and Uylings, 2002; Stiefel and Sejnowski, 2007; Torben-Nielsen and Cuntz, 2014). In this approach virtual morphologies are generated *de novo* using morphogenetic algorithms. In most cases, these algorithms adhere to the ideas proposed by Hillman and sample from statistical distributions representing morphometric features to generate a morphology (Eberhard et al., 2006; Lindsay et al., 2007; Torben-Nielsen and Cuntz, 2014). Clearly, these methods can mimic statistical properties of the data set but fail to capture contextual influences and plausible variation (but see Samsonovich and Ascoli, 2003). Notable exceptions exist and target specific characteristics of the context. Luczak proposed a generative method based on diffusion-limited aggregation to illustrate how competition over resources and the spatial distribution thereof could shape dendritic morphologies (Luczak, 2006). In another work, Cuntz and colleagues proposed a generative approach based on high-level wiring constraints. By generating multiple virtual morphologies in the same volume, competition over resources could be mimicked (Cuntz et al., 2010). In previous work, we demonstrated that self-referential contextual cues (e.g., self-avoidance, soma-tropism, and membrane stiffness) could be used to explain some characteristics of dendritic morphologies (Memelli et al., 2013). Recently, CX3D was designed to simulate neuronal development based on intrinsic and extrinsic, contextual factors (Zubler et al., 2013).

In this work we argue that in order to fully understand neuronal morphologies we need to break with the view that neurons can be treated as independent, isolated entities. Therefore, we propose a new approach to study morphologies in which large numbers of virtual morphologies are generated simultaneously *de novo* while embedded in a virtual brain substrate, resulting in a mechanistic – in contrast to a statistical – description of morphologies. In this approach, morphologies are generated by repeatedly extending simulated, phenomenological growth cones that are guided by interactions with other actors in the brain substrate.

We designed and implemented a prototype of the proposed computational framework, NeuroMaC (“Neuronal Morphologies and Circuits”). We showcase the functionality of our framework related to single neuron morphologies by synthesizing spinal cord motor neurons, hippocampal granule cells and cortical layer 5 (L5) pyramidal neurons. All results are validated against publicly available, experimentally reconstructed morphologies.

## MATERIALS AND METHODS

### OUTLINE

The rationale behind our proposed framework is based on two key experimental findings. The first is that the genetic make-up of a neuron determines its shape to a large extent. In cell culture experiments, neurons have a recognizable morphology, albeit one that differs from *in situ* occurrences (Banker and Cowan, 1977; Kriegstein and Dichter, 1984). Second, the genetic make-up of neurons also appears to outline a blueprint of neurons in terms of interactions with the substrate in which they develop. Growth is mainly determined by growth-cones that contain filopodia-like structures that sense the molecules present in the extracellular matrix. Sensation of these molecules then influences when a growth cone branches or terminates as well as the direction of elongation (Itoh et al., 1993; Scott and Luo, 2001; Mortimer et al., 2008; Jan and Jan, 2010).

We extrapolate these key findings to operational concepts in our framework that simulates phenomenological growth cones called fronts. Broadly speaking, fronts contain growth rules that can be expressed in terms of interactions with other agents present in the substrate. Interactions are always “local” in the sense that a front is able to sample its direct surrounding. As such, fronts are a simple metaphor for biological growth cones.

**FIGURE 1:**
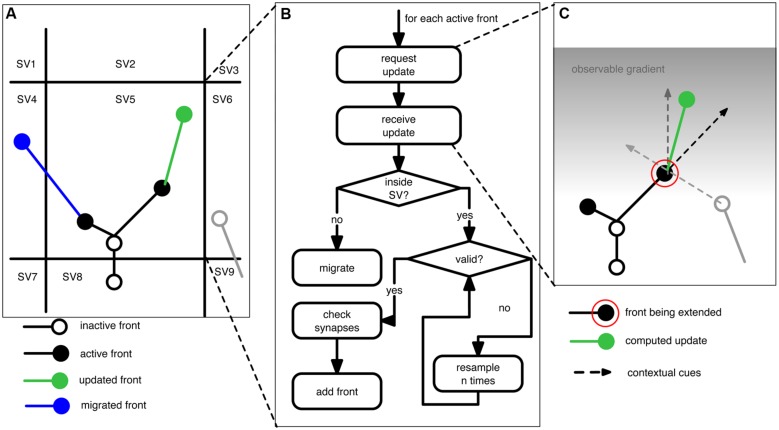
**Schematic of the proposed context-aware framework, NeuroMaC, to generate virtual morphologies. (A)** The simulated brain substrate is decomposed into small sub volumes (SVs). Sub volumes keep track of all neurites and other relevant actors inside their spanning volume. **(B)** Algorithm performed by each sub volume during one simulated, centrally controlled time step. **(C)** Fronts are implemented as cellular automaton-like machines and conceptually related to growth cones in that they update their location based on the local context. Full lines: neurites (black and gray: existing; green: newly added). Circles represent active (filled) or inactive (open) fronts. Dashed lines represent the contextual cues influencing the direction of growth of an active front to be extended (indicated by a red circle). Here the contextual cues are defined by an inertial forward-directed influence, another neurite, and a gradient in the substrate.

**Figure 1** outlines the concepts underlying NeuroMaC. Based on the “local” nature of sensing and sampling of fronts we can decompose the simulated brain volume into small sub volumes (SVs). Each SV has full knowledge about all contained fronts and contextually relevant actors in the substrate, e.g., boundaries and other neurons amongst others. All SVs repeatedly extend all active fronts contained inside their spanned volume. Because fronts also have a physical dimension with a location and a radius, extending fronts creates the simulated neurites by creating a frustum between the initial position of a front and the new position after extension. Details about the construction rules of fronts are provided in the next section and for now it suffices to understand that – in line with the behavior of growth cones – fronts can extend, branch or terminate, and that they can use contextual cues to influence these actions. Once the active fronts are extended, the SVs perform the crucial step of checking and resolving structural overlaps while simultaneously recording locations of putative synapses. As a result, generation of morphologies and construction of a circuit (without structural overlaps) can be performed in one pass.

### NeuroMaC

We designed and implemented NeuroMaC in accordance to the rationale and key concepts outlined above. Here we describe in-depth the components of the proposed framework.

#### Multi-agent architecture and parallelization

NeuroMac is designed as a multi-agent system, that is, different components of the framework work autonomously and communicate with each other through messages. A multi-agent system allows straightforward parallelization with the number of computing cores to ensure scalability. NeuroMaC has two agent types: one central administration agent and multiple SV-agents.

The administration agent performs all internal housekeeping. It reads a configuration file (**Table 1**) that defines the simulation and system specific settings. Subsequently the administration agent decomposes the brain substrate into smaller SVs and initializes the SVs. During initialization each SV is assigned a space it controls together with all environmental details required for the fronts to develop. The administration agent maintains a central clock to synchronize updating of fronts in each SV. A clock ensures that irrelevant issues such as execution time on the computing resource do not bias simulated growth. In case an updated front moves outside of the space covered by a particular SV, the administration agent brokers the migration of that front to the appropriate new host SV. All updates inside an SV are communicated to the central agent, which compiles a centralized output file containing all neuronal morphologies.

**Table 1:** Exemplar configuration file used in NeuroMaC.

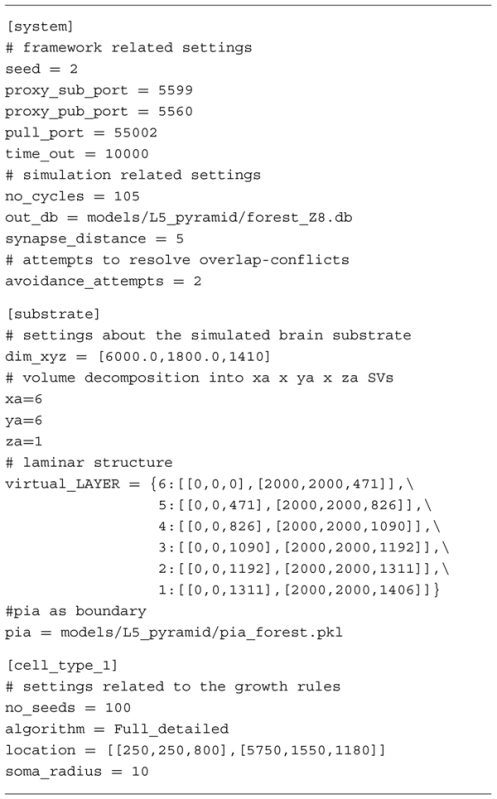

*The configuration follows the Python ConfigParser structure. Parameters are pooled in several sections and parameter values can take the form of executable Python statements. A description is in the main text.*

The SV agents perform the same behavior in parallel. The number of these agents can scale with the number of available computing nodes; more nodes results in smaller decomposed volumes and faster run times. Conceptually, SVs represent the direct neighborhood surrounding a developing growth cone. Distal parts of the brain substrate are of no concern to a growth cone as all contextual cues are sensed in the direct vicinity. SVs contain all local information about the substrate itself, e.g., boundaries, laminar structure, same and other neuron structures, etc. Diffusible molecules in the extracellular space can promote long-distance interactions and while we do not simulate diffusion explicitly, the effect of contextual cues can propagate from SV to SV so that these are also locally available for growth cones. Any cue not on the hosting SV or on one of the direct neighbors is summarized (averaged) and only this information is revealed to active fronts. This measure is valid because it is irrelevant for an active front to know the exact locations of very distant cues.

During each general time step SVs execute the algorithm listed in **Figure 1B**. However, just before the algorithm is executed, each SV communicates with its neighbors to query their contained volume. This is needed because, if an active front is close to an SV boundary (e.g., close enough that it might interact with a neurite contained in a neighboring SV), it also has to sense the neighboring substrate. During the main algorithm, SVs call each active front inside their volume, in randomized order, to compute its next location (see next section). Once the SV receives the updated front, it performs several checks. First, it checks if the new location of the front is still inside the volume it spans. If not, the front is migrated to another SV. Otherwise, the SV checks whether the new front physically overlaps with existing fronts and neurites. Overlap is tested between two fronts and their associated line segments. That is the line segment between a front and its parent. If the minimal distance between two such line segments is smaller than the sum of the radii of both associated fronts we consider this to be an overlap. Unless the radius of a front is drastically smaller than that of its parent front, this method yields adequate results. When a potential overlap is detected, the SVs will try to resolve it by randomly perturbing the front’s location. If the conflict cannot be resolved in a predetermined number of attempts, the front is terminated at its previous position. When all active fronts are updated and validated, the corresponding newly formed neurites are communicated to the administration agent. Putative synapse locations are computed in the same way (and at the same time) as the structural overlaps with the difference that a maximally allowed distance is set by the user that reflects the pre-synaptic bouton and post-synaptic spine size. Although rudimentary, this method yields a list of putative synapse locations that can be pruned in a post-processing step (Hill et al., 2012), but also see van Pelt et al. (2010).

#### Growth cones as cellular automata

In NeuroMaC fronts are phenomenological implementations resembling biological growth cones. An active front is a front that is still developing; an inactive front becomes continuation point, branching point or a terminal tip. As such, neurites are represented by frusta connecting subsequent fronts (**Figure 1C**; Cannon et al., 1998; Ascoli et al., 2007).

Fronts have a dual identity. On the one hand they are physical structures with a location and radius in space. On the other hand, a front is a cellular automaton-like machine that contains its own growth rules describing how and when it should extend, branch or terminate (see **Table 2** for an example). When an active front is not terminating, it either produces one or two new fronts; the old front becomes inactive and the newly formed front(s) become(s) active fronts. The location of the new front is computed in accordance to a front’s construction rules and locally available information. Information can be everything that is contained in the SV. For instance, homotypic (Grueber et al., 2005; Marks and Burke, 2007; Memelli et al., 2013) and same-type (Scott and Luo, 2001; Jan et al., 2003) cues can be used, or, the transient laminar information through which a front might travel (Hevner et al., 2003; Chen et al., 2005). The aforementioned cues have a direct biophysical interpretation, but also more phenomenological cues such as (directional) information related to a boundary can be used in our framework. A biological counterpart thereof could be envisioned to be Reelin secreted by Cajal-Retzius cells (Frotscher, 1998; Marin-Padilla, 1998). Construction rules define how the front interacts with these other inhabitants of the SV: no interaction, repulsion or attraction. Hence, the context is used as a guidance cue (**Figure 1C**). The influence of these cues can be distance-dependent mimicking gradients of secreted molecules (Mortimer et al., 2008). In addition, fronts can also modify the substrate by secreting entities: phenomenological representations of secretion molecules that can in turn be used as a guidance cue (Hentschel and van Ooyen, 1999).

**Table 2:** Complete Python code used to implement the growth rules underlying the generated motor neurons (illustrated in **Figure 2**).

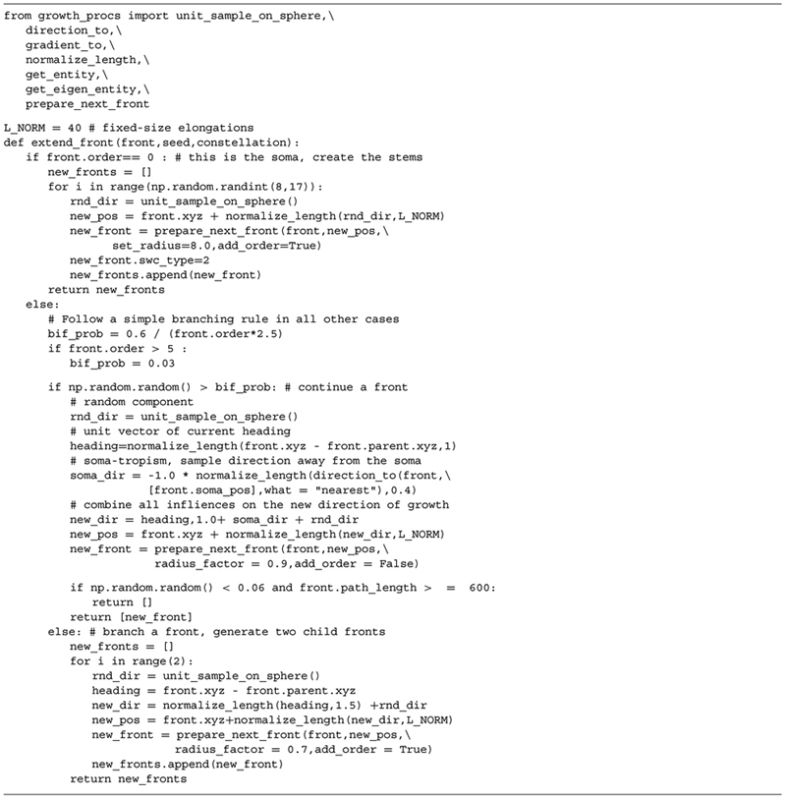

*NeuroMaC contains auxiliary function to build fronts and sample the context; these functions are first imported. The main function “extend_front” is called by the sub volume and contains the actual growth rules. In this example, a single contextual cue, soma-tropism, is used.*

### IMPLEMENTATION

We implemented a prototype in Python and use ZeroMQ (Hintjens, 2013) to send messages between the components because it has the ability to buffer large messages and operate asynchronously. The algorithm underlying the behavior of an active front is a Python script and is the only part that has to be implemented by an end-user. This prototype is available on https://groups.oist.jp/cnu/neuromac.

Combined, the eminent features of NeuroMaC are: (1) Context-aware generation of virtual morphologies that will not overlap with one another in space; (2) The ability to detect and record synapses on the fly; and (3) Straightforward scalability and parallelization to generate large numbers of morphologies at the same time.

## RESULTS

In order to validate the proposed framework we generated sets of virtual neuronal morphologies and compared them to the statistics of experimentally reconstructed morphologies. We validate NeuroMaC by demonstrating that we can (1) generate morphologies in isolation as current state of the art approaches do, (2) populate a space by generating a forest of non-overlapping and interacting hippocampal granule cells, and (3) generate fully context-aware morphologies that interact with the environment (L5 pyramidal neurons in a laminar architecture). We selected these neuron types because motor neurons and hippocampal granule neurons are often used in algorithmic generation; pyramidal neurons were chosen because their higher morphological complexity and assumed context-dependence. The experimentally reconstructed neurons were downloaded from NeuroMorpho.org (Ascoli et al., 2007). We took two motor neuron archives, the Burke archive (*N* = 6, Cullheim et al., 1987) and the Fyffe archive (*N* = 8, Alvarez et al., 1998). The granule neurons come from the Lee archive (*N* = 25, Carim-Todd et al., 2009). Pyramidal neurons are layer 5, secondary motor cortex neurons and come from the Kawaguchi archive (*N* = 10, Hirai et al., 2012).

### MOTOR NEURONS IN ISOLATION

**FIGURE 2:**
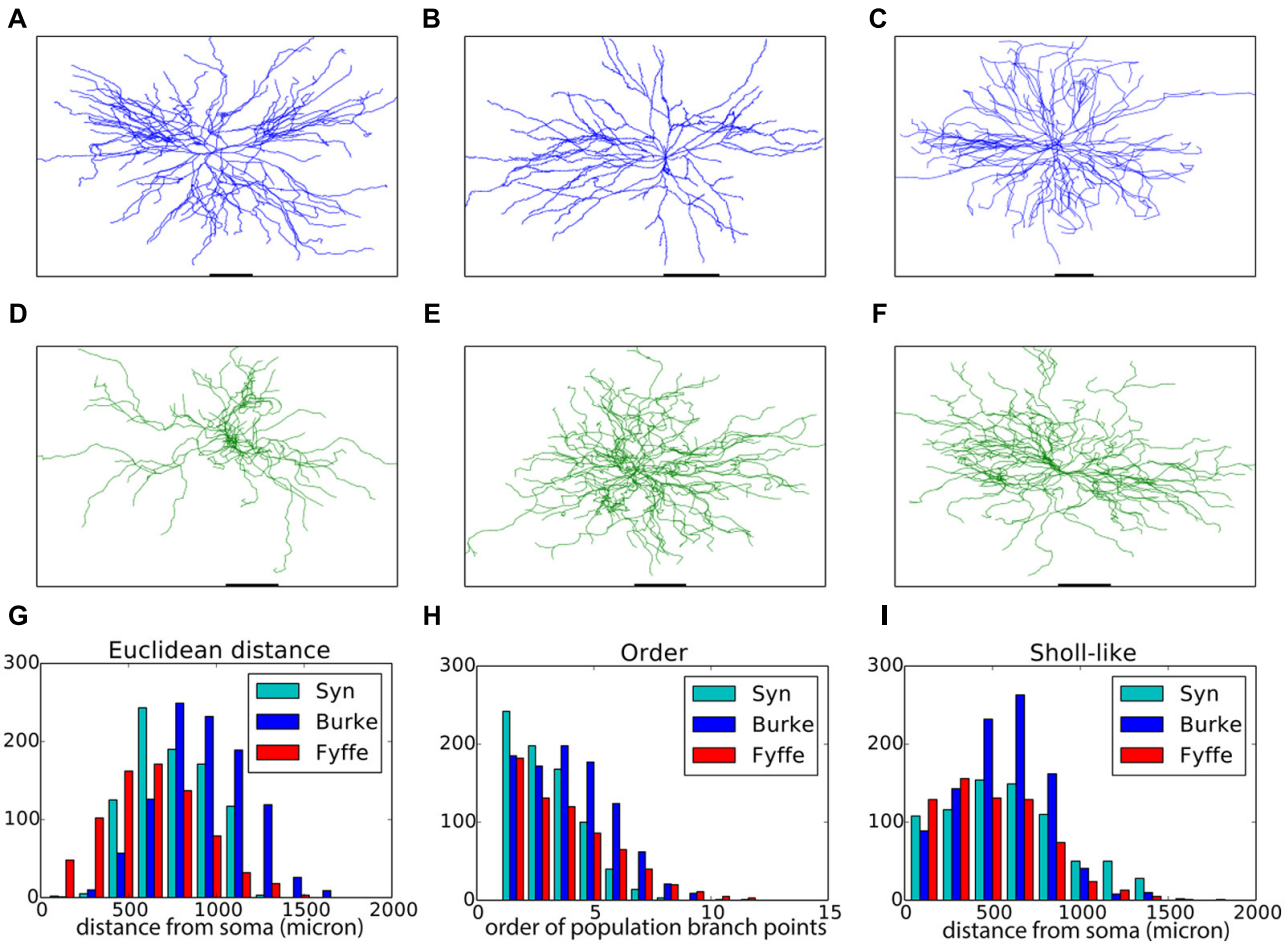
**Validation of generated alpha motor neurons. (A–C)** Exemplar experimentally reconstructed spinal cord alpha motor neurons [**A,B** from the Fyffe archive (Alvarez et al., 1998), **C** from the Burke archive (Cullheim et al., 1987)]. **(D–F)** Virtual morphologies generated by NeuroMaC. **(G–I)** Quantitative comparison. Population morphometrics are shown for the Burke (“Burke”) and Fyffe (“Fyffe”) archives and for the generated morphologies (“Syn”). **(G)** Euclidean distance between the soma and each terminal point in all morphologies. **(H)** Topological order of each branching point in all morphologies. **(I)** Occurrence of branching points in each morphology as a function of Euclidean distance (i.e., Sholl-intersections, see main text). See **Table 3** for detailed statistics of these (and other) morphometrics.

Motor neurons have a relatively straightforward morphology that, from the point of view of an external observer, is fairly context-independent (**Figures 2A–C**). We devised a purely phenomenological growth rule to mimic the final morphology consisting of two sub rules: one rule for the initial front (=the soma) and one rule for all other fronts. The full Python code of the growth rule is listed in **Table 2**. At the soma (“front.order == 0”), multiple stems are created in random directions around the soma. Once the stems are created fronts can bifurcate with a probability inversely proportional to the branching order, terminate with a small probability or extend otherwise. When a front grows outside the assigned substrate space it is terminated. Current heading, repulsion by the soma and a random component set the direction of a bifurcating or extending front. Typical resultant virtual morphologies are listed in **Figures 2D–F**.

Visual inspection shows high resemblance between the exemplar and generated motor neuron morphologies. We then checked the global morphometric, namely the Euclidean distance between the soma and terminal tips (**Figure 2G**) and the two-dimensional local metrics “order” that expresses the occurrences of branching points as a function of branching order (**Figure 2H**), and, “Sholl-like,” a quick implementation of the Sholl metric that measures branch points as a function of Euclidean distance from the soma (**Figure 2I**). Trends contained in the experimentally reconstructed neurons (labeled “Burke” and “Fyffe”) are replicated by the generated neurons (labeled “Syn”). We quantify the distribution by the median (M) and median absolute deviation (MAD) because the shape of the resultant distribution of the measures is unknown a priori and do not necessarily follow a normal distribution. Spread of the distribution is quantified with the interquartile range (IQR). Quantification is listed in **Table 3**. From the quantification we can see that there is a fair difference between the exemplar archives and that the generated neurons fit well between the values of the exemplars.

**Table 3:** Quantification of generated and experimentally reconstructed alpha motor neurons.

		Synthetic	Burke	Fyffe
# branch points	M	125	161	55
	MAD	11	12.5	20
	IQR	41	23.7	82
Euclidean D	M	750	926	616
	MAD	171	184	277
	IQR	352	372	380
Max order	M	7	9	7
	MAD	0	0	1
	IQR	1	0	2.5
Order	M	3	4	4
	MAD	1	1	1
	IQR	2	2	3
Sholl-like	M	600	575	445
	MAD	240	171	213
	IQR	480	350	435
Total length	M	69,674	105,373	28,876
	MAD	14,041	8730	14,691
	IQR	24,220	14,169	41,363

Distribution of observed morphometrics are given by the median (M), median absolute deviation (MAD) and inter-quartile range (IQR). Values shown for the generated (“synthetic”) morphologies and the morphologies originating from the Burke and Fyffe archives (see main text).

Both visual inspection and the quantitative measures show a good correspondence between the experimentally reconstructed and generated morphologies. These results are on par with the previously published results (Memelli et al., 2013), and hence we can conclude that by using NeuroMaC we can create sets of neurons generated in isolation.

### A FOREST OF HIPPOCAMPAL GRANULE NEURONS

**FIGURE 3:**
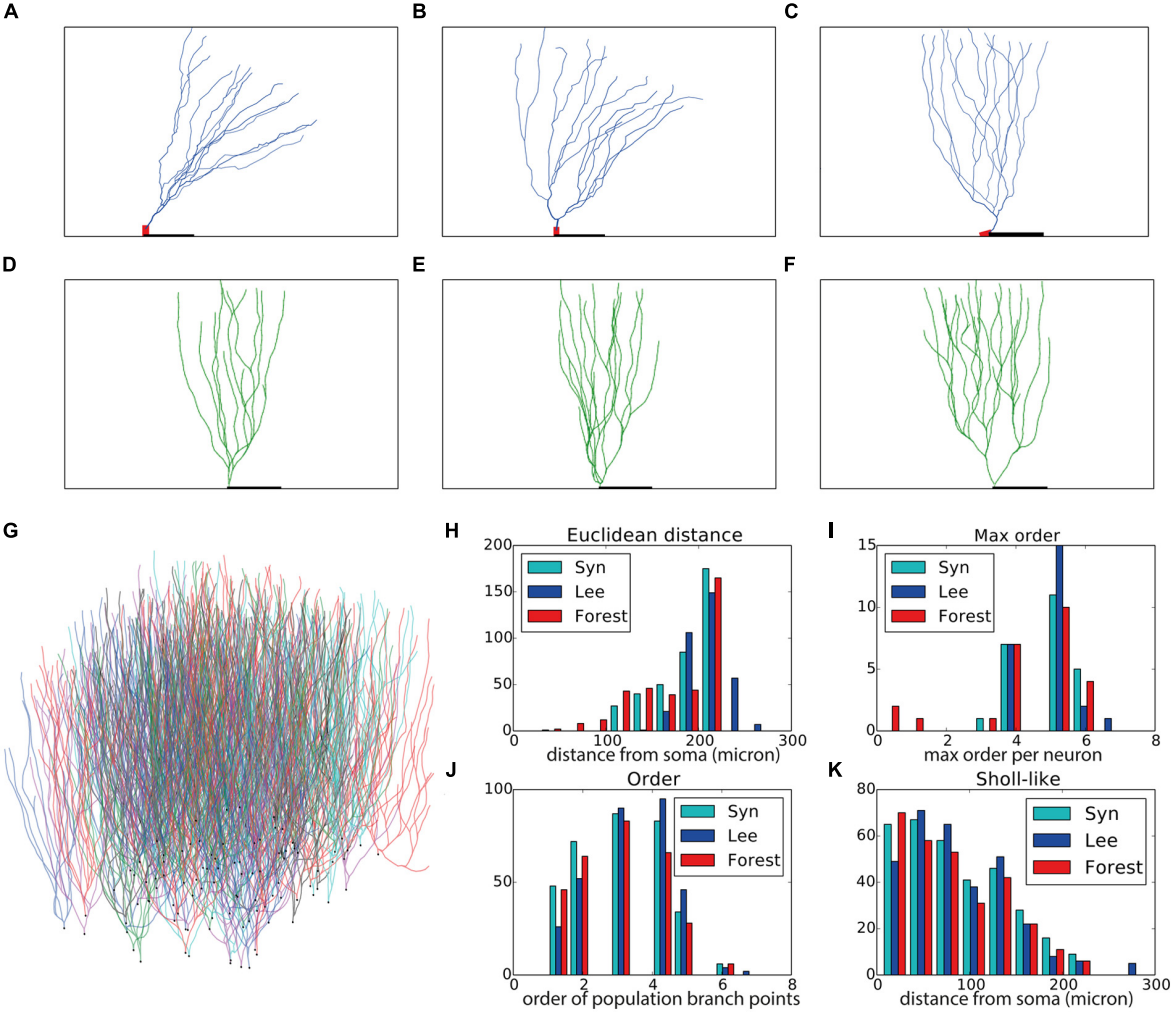
**Validation of generated hippocampal granule cells. (A–C)** Experimentally reconstructed granule cells (from the Lee archive; Carim-Todd et al., 2009). **(D–F)** Virtual morphologies generated by NeuroMaC. **(G)** Forest of 100 simultaneously generated, non-overlapping granule cells. **(H–K)** Quantitative comparison. Population morphometrics are shown for the Lee archive (“Lee”), synthetic neurons generated in isolation (“Syn”) and as part of a forest (“Forest”). **(H)** Euclidean distance between all terminal tips and the soma. **(I)** Maximum topological order in the individual morphologies. **(J)** Topological order of each branching point in all morphologies. **(K)** Occurrence of branching points in each morphology as a function of Euclidean distance (i.e., Sholl-intersections). See **Table 4** for a detailed quantification of these (and other) morphometrics.

Next we set to generate granule cells, both in isolation and in a “forest” setting, that is, many neurons packed in one volume with all neurons being generated simultaneously. Three experimentally reconstructed exemplar morphologies are shown in **Figures 3A–C**. We devised a straightforward construction rule in a similar vein to the rule used for the virtual motor neurons. Once the soma and two initial branches are created, branching occurs with a probability that decreases with the centrifugal order of the front. The direction of growth is determined by repulsion away from same-neuron dendrites, the current heading of a dendrite, and the direction towards the superficial part of the substrate, which in this case is the superficial part of the dentate gyrus. A random component is added to all growth directions as well. We generated two sets of virtual morphologies, namely a set in which each neuron was generated in isolation (*N* = 25, **Figures 3D–F** are representative examples) and one set in which 100 morphologies were generated simultaneously in a (**Figure 3G**). The growth instructions were kept identical in both sets. The simulated volume, however, was increased 20-fold in the forest setting (i.e., 1300 μ × 300 μ × 225 μ, with 225 μ being a plausible depth of the dentate gyrus). Note that in the “forest” setting, developing morphologies interact indirectly with each other through overlap-prevention.

Visually the generated morphologies bear strong resemblance to the exemplar ones. We then measured the Euclidean distance between some and terminal tips and the maximum order in a tree (**Figures 3H,I**), as well as the two-dimensional “Order” and “Sholl-like” metric (**Figures 3J,K**) for the set of exemplar morphologies (“Lee”) and the sets of morphologies generated in isolation (“Syn”) and in a forest setting (“Forest”). To avoid biases introduced to an unequal number of samples, we randomly picked 25 morphologies from the forest and computed the appropriate features from this subset. The histograms indicate similar trend in the data of all data sets. Quantification of all measured morphometrics is provided in **Table 4**. It is interesting to note that the variance in the morphologies generated in a forest setting is higher. This observation results from the fact that all these neurons are generated simultaneously. As a result, some branches would overlap with each other. Overlaps are detected and an attempt is undertaken to resolve the overlap. However, if no quick resolution is found, the branch is terminated. In the forest setting, the somata are close to each other and some conflicts in the proximal branches could not be resolved and caused very small Euclidean length and low maximal order in rare cases (**Figures 3H,I**, left-most red bars). The two dimensional metric indicate a good match in the topological and geometrical distribution of branch points (**Figures 3J,K**).

**Table 4:** Quantitative description of experimentally reconstructed hippocampal granule neurons and their generated counterparts.

		Lee	“Isolation”	“Forest”
# branch points	M	13	12	13
	MAD	1	4.5	5
	IQR	2	8.25	9
Euclidean D	M	207	199	197
	MAD	12.7	11.7	16.7
	IQR	26.2	41.7	68
Max order	M	5	5	5
	MAD	0	1	1
	IQR	1	1	1
Order	M	3	3	3
	MAD	1	1	1
	IQR	1	1	1
Sholl-like	M	77	79	75
	MAD	38.5	40.5	40.1
	IQR	84	85	80
Total length	M	2255	1846	1590
	MAD	258	437	505
	IQR	391	1001	922

*Generated morphologies can be generated in isolation or in a forest setting. Presentation as in **Table 3**. Values shown for the morphologies from the Lee archive and the morphologies generated in “Isolation” and in the “Forest” settings (see main text).*

Even though the neurons in the forest setting were densely packed (**Figure 3G**) no overlaps occurred as neurite locations were either corrected or terminated during the validity checks performed by the SVs. Therefore, we conclude that with NeuroMaC we can generate forests of non-overlapping, plausible morphologies.

### CONTEXT-AWARE L5 PYRAMIDAL NEURONS

**FIGURE 4:**
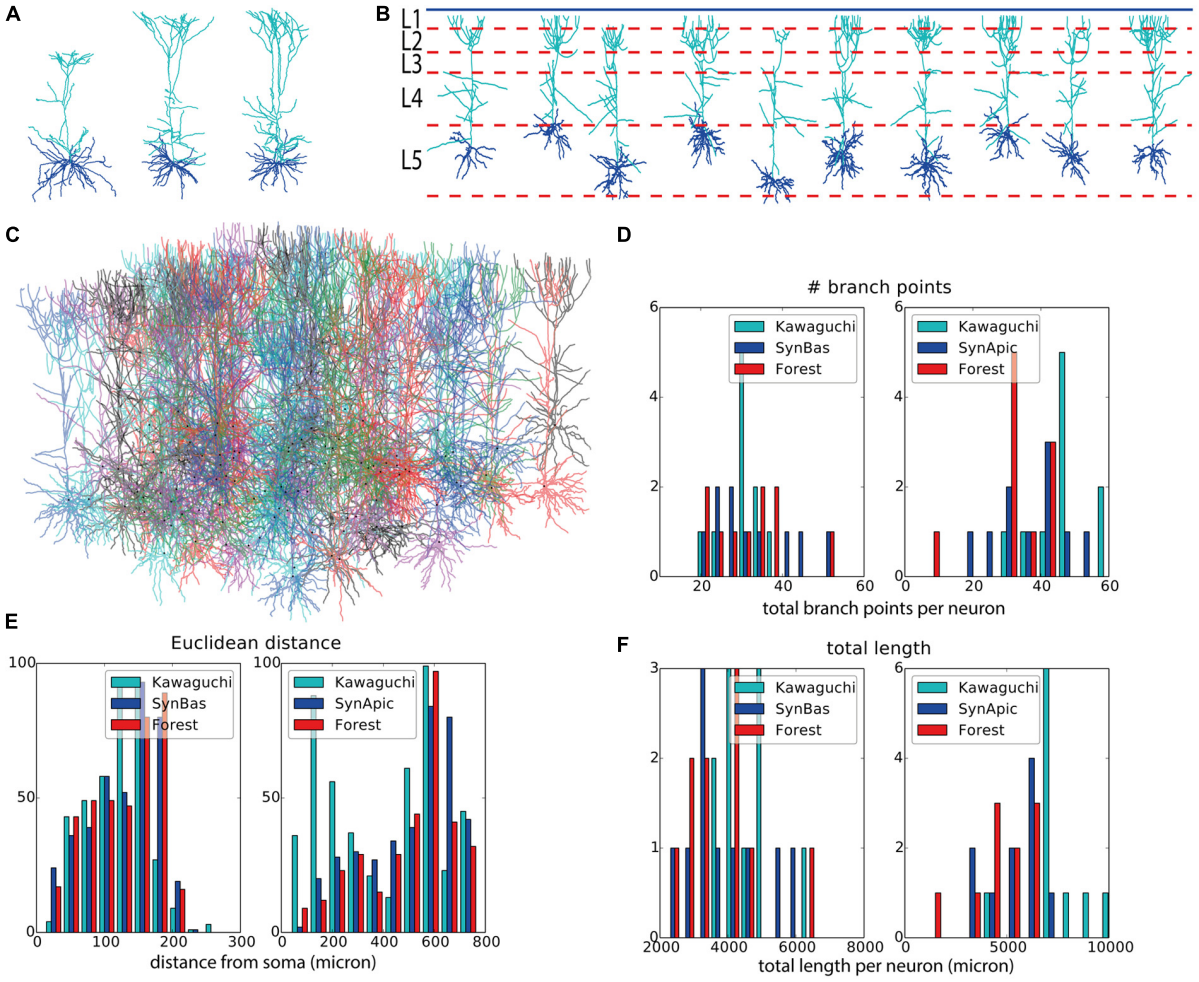
**Validation of generated layer 5 pyramidal neurons. (A)** Experimentally reconstructed layer 5 pyramidal neurons (from the Kawaguchi archive). **(B)** Virtual morphologies generated by NeuroMaC. Simulated laminar structure (L1–L5, from top to bottom) indicated by dashed lines; blue line represents the pia. **(C)** Forest of 100 simultaneously generated, non-overlapping pyramidal neurons. **(D–F)** Quantitative comparison. Population morphometrics are shown for the Kawaguchi archive, synthetic neurons generated in isolation (“Syn”) and as part of a forest (“Forest”). Statistics are given for basal (left panels) and apical (right panels) trees separately. Shown are total number of branching points **(D)**, Euclidean distance between terminal tips and the soma **(E)** and the total length of the dendrites **(F)**. Detailed statistics of these (and other) morphometrics in **Table 6**.

As a final demonstration of the capabilities of NeuroMaC, we generated context-dependent layer 5 pyramidal neuron morphologies. Three exemplar morphologies are shown in **Figure 4A**. By visually examining these morphologies, we can observe some morphological traits such as a difference in “height” but these traits are hard to relate to their context. However, from canonical circuit information, we know that the somas are located in layer 5, that their basal dendrites remain mainly in L5 and may extend a bit into L4, that their apical dendrite extends to the superficial parts and ends close to the pia (in L1) after branching extensively in layers L3–L1, and, that oblique dendrites sprout from the apical trunk in L4. The remarkable difference in “height” of the apical tree, is a clear signature of this context dependence as more superficially located pyramidal cells cannot extend as far as more deeply positioned ones.

We designed construction rules that take these canonical, contextual traits based on laminar structure into account. A truncated code snippet is listed in **Table 5** to indicate particular context-dependent growth rules. Note that the growth rules are different for basal and apical dendrites, and a further division of the apical growth rules into rules for L5/L4, oblique dendrites, and the dendrites in L3/L2/L1. At the soma, we generate an appropriate number of basal stems and one apical stem. The basal dendrite branches with a probability inverse proportional with the centrifugal order; at orders higher than 6 no branching is allowed. Termination of a basal branch occurs with a small probability or when a branch grows outside the limiting volume. Direction of growth is again influenced by the heading and same-neuron repulsion and an additional random factor. The apical branch is contextually aware and the construction rules change depending on the layer it is in (**Table 5**, “extend_apical_front”). Layer-dependent behavior is biologically feasible because in cortex some transcription factors are exclusively expressed in layer specific neurons (Hevner et al., 2003; Chen et al., 2005). In layers 5 and 4, oblique dendrites can sprout and grow away from their initial branch point at the apical trunk. In subsequent layers (3, 2, and 1) neurons can branch with layer specific probabilities as long as a maximum increase in order has not occurred yet in one layer. Same-neuron repulsion, current heading, a distance-dependent attraction to the pia, and a random component determine the direction of growth in the superficial layers 3–1. Apical neurites can terminate as soon as they reach layer 3 (and later 2 and 1) with a small probability. All apical neurites are terminated if the pia is closer than 35 μ away.

**Table 5:** Code snippet illustrating the growth rules to generate layer 5 pyramidal neurons.

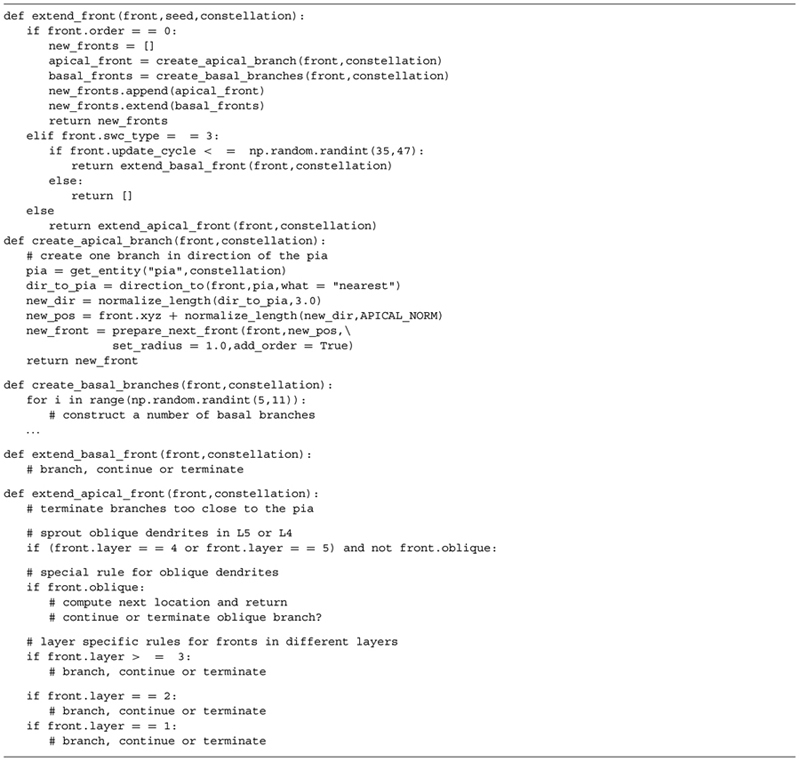

*The code is incomplete and merely for the purpose to illustrate some of the context-dependent cues such as growth direction to the pia and layer specificity (for the apical tree).*

Two sets of morphologies are generated; again one with neurons in isolation (*N* = 10 to match the sample size in the Kawaguchi archive) and one with 100 simultaneously generated morphologies in a forest setting. The volume in the “forest” setting was a rectangle of size 6000 μ × 1800 μ × 1400 μ, where 1400 μ is the estimated depth of L5 in the exemplar data. All morphologies from the former set are plotted in **Figure 4B** along with the canonical virtual laminar architecture in which they grew (blue line: pia, red dashed lines: layer boundaries. Layer 1 is at the top and layer 5 at the bottom; layer 6 is not shown). The forest from the latter set is plotted in **Figure 4C**.

Visually, the generated neurons clearly exhibit the morphological traits summarized above. Furthermore we compared the total number of branch points (**Figure 4D**), the Euclidean distance to the terminal tips (**Figure 4E**) and the total length (**Figure 4F**). A quantification of all measured morphometrics is listed in **Table 6**. The basal and apical dendrites are treated separately in these measures. The basal trees show great correspondence with the exemplar morphologies in terms of the Euclidean distance to the terminal tips and the total length of the dendritic trees. The number of branch points in the generated neurons is markedly higher than in the exemplar ones; a range of [19,39] for the Kawaguchi archive and [20,52] and [19,53] for the generated neurons in isolation and forest setting, respectively. Given a correct match with the total length and the Euclidean distance to the tips, we speculate that the simple branching and termination rules are not sufficient for the basal trees, although the low number of branch point can also result from incomplete reconstructions (Anwar et al., 2009, but also see Section “Discussion”).

**Table 6:** Quantitative description of experimentally reconstructed L5 pyramidal neurons and their generated counterparts.

			Kawaguchi	“Isolation”	“Forest”
# branch points	apical	M	46	40.5	33
		MAD	3	10	5
		IQR	4.7	13.7	10
	basal	M	31	30	32
		MAD	1.5	6	5.5
		IQR	2.5	14.2	10
Euclidean D	apical	M	407	561	552
		MAD	198	116	98.3
		IQR	409	265	237
	basal	M	125	138	137
		MAD	28.5	35.7	38.8
		IQR	62.1	76.7	85.9
Max order	apical	M	17.5	19	18
		MAD	2.5	1	2
		IQR	4.7	2.5	4.7
	basal	M	5	5	5
		MAD	1	0	0
		IQR	1.7	0	0
Order	apical	M	10	16	15
		MAD	5	3	3
		IQR	10	8	8
	basal	M	2	3	3
		MAD	1	1	1
		IQR	1	2	2
Sholl-like	apical	M	345	521	554
		MAD	224	171	131
		IQR	448	378	322
	basal	M	41	70	70
		MAD	13.3	35	42
		IQR	28.3	77	84
Total length	apical	M	7327	5645	4882
		MAD	489	637	853
		IQR	846	1398	1568
	basal	M	4439	3398	3664
		MAD	518	672	1461
		IQR	945	1461	1263

*Generated morphologies can be generated in isolation or in a forest setting. Basal and apical dendrites are treated separately. Presentation as in **Table 3**. Values shown for the morphologies from the Kawaguchi archive and the morphologies generated in “Isolation” and in the “Forest” setting (see main text).*

Considering the apical trees, we observe a mismatch in the Euclidean distances and the total length between the exemplar and the generated morphologies. We attribute both to a difference in the oblique dendrites. As seen in **Figure 4E** (left panel, “Kawaguchi”), there is a peak of terminals in the apical dendrite that terminate close to the soma. While the generated data also displays a second peak due to terminals of the oblique dendrites, this peak is less pronounced and shifted to greater Euclidean distances. We speculate that in the exemplar dendrites, more oblique dendrites sprouted more proximally than in our model. Given a major thalamic synaptic pathway in cortex projecting to layer 4 and synapsing onto oblique dendrites (Meyer et al., 2010; Oberlaender et al., 2012), it is not unreasonable to think the oblique dendrites mainly sprout in layer 4 as in our model. But, as said, an SWC file does not contain any contextual information so the true dimensions of the laminar architecture of the animals from which the neurons were reconstructed remain a guess. Moreover, we consider the ability of NeuroMaC to construct context-dependent dendrites a quality, even if no context-dependent information related to the exemplar morphologies was directly available. The fact that the apical trees generated by NeuroMaC all reach the L1 – and not further – are a great illustration of this context-dependence.

Our results indicate a clear and valid context-dependence, which is similar to the morphological traits in the exemplar data. Therefore, we can conclude that the generated morphologies exhibit context-dependent morphological traits that match to the traits discovered in the exemplar data.

## DISCUSSION

We started this work with the observation that there is a large discrepancy between the way neuronal morphologies are studied (in isolation) and the way they develop and take their shape (in interaction with a dense surrounding substrate). From experimental studies it appears that the surrounding brain substrate, the context of all neurons, plays a pivotal role in shaping the morphology and resultant brain circuits. To overcome this discrepancy, we proposed a new computational framework, NeuroMaC, to study how neuronal morphologies emerge from interactions with other actors in the brain substrate.

We opted for a phenomenological framework for the sake of conceptual simplicity and to curb computational costs. Construction rules are conceptually related to the genetic make-up of a neuron and express how a neuron has to grow in terms of repulsive or attractive interactions with the surrounding substrate. A phenomenological framework helps to reduce the computational resources in contrast to biologically and physically detailed ones. Moreover, the design of NeuroMaC as a multi-agent system ensures scalability with the number of available processors. As a consequence of the design choices, NeuroMaC can be used to generate large numbers of interacting morphologies simultaneously. This feature is unrivaled. CX3D, an existing computational tool aims to simulate the whole of cortical development, from migration over polarization and differentiation to dendrite and axon formation. However, the main version is serial (i.e., not parallel) which limits its applicability to generate multiple full morphologies at the same time. NETMORPH, a tool capable of generating large cortical networks (Koene et al., 2009) adopts a strategy in which a volume is populated by adding neurons that are generated in isolation. The topology of neurons is based on a mechanistic growth rule but the geometry assigned to embed the topology in space is statistically sampled from exemplar data. Hence, in NETMORPH all neurons are independent and not based on any contextual cues (van Ooyen et al., 2014). Although it has to be noted that exemplar data contains morphologies that are shaped through contextual interactions and, therefore, if a model succeeds in reproducing morphological traits it implicitly captures some of these interactions. Historically, ArborVitae (Senft and Ascoli, 1999) was proposed to generate large networks of neurons simultaneously and with some phenomenological interaction based on resource competition. While promising initial results were generated, this tool is no longer in development. Hence, NeuroMaC is currently the only computational framework to study explicitly how neurons grow together while interacting with the environment.

We demonstrated that by using NeuroMaC we can generate plausible neuronal morphologies with construction rules based on local interactions, which inhabit the same simulated substrate and have no physical overlaps. In the current work, construction rules underlying the growth of morphologies are a crude approximation of the hypothesized growth rules used by neurons. The aim of this work was not so much the generation of the most “realistic” morphologies or morphological traits but rather showcasing the power and usability of our new framework. As such, we illustrated that construction rules expressed in terms of repellants and attractors are a useful metaphor to study morphologies.

NeuroMaC can be used in any desired way on the continuum between small and large spatial scales and their associated level of biological detail. At one end of this continuum it can be used to study the effects of detailed, biologically plausible construction rules. This way, studies can be conducted investigating how particular construction rules representing biophysical processes influence morphological traits. On the other end of the continuum, one could opt to use less detailed rules to generate full morphologies and, because putative synapse locations are recorded as well, the resultant circuits. Of course, highly detailed construction rules can also be used (at little extra computational cost) to generate full circuits and any “intermediate” level of detail can be implemented as well. However, while it is possible to compute the propagation of microscopic rules to the meso-scale circuit, it can be a tedious task to analyze the whole circuit at large for traces of the underlying microscopic interactions. Another noteworthy feature of NeuroMaC is that it supports a mixed-methodology with respect to the growth rules. That is, existing context-independent neurogenetic algorithms can be implemented in a straightforward fashion so that they can be used as growth rules. As such, a simulated brain substrate could be populated by morphologies grown in accordance to different methodologies.

One important observation is that our virtual morphologies generated in a forest setting exhibit a larger variance than present in the exemplar data (**Figures 3I,J** and **4D,F**). This effect is smaller but still present in the neurons generated in isolation. We turn to the data sets of experimentally reconstructed neurons to explore the issue of variance.

We can start by assuming that the data is a good representative of all neurons. In that case, our data exhibits too much variation. Here the explanation would be that the used branching rules are too simple and that branch probability and termination are also dependent on both intrinsic and extrinsic signals. Intrinsic signals could be mediated through the production and transport of actin filaments that are required for scaffolding the neuronal membrane (Graham and van Ooyen, 2004). A detailed, mechanistic rule based on these intrinsic properties has been proposed (van Pelt and Verwer, 1986; van Pelt and Schierwagen, 2004) and could be used in our framework. Extrinsic signals are inherently context-dependent. Experimental work has demonstrated that the presence of specific molecules in the extra-cellular space influence branching and termination properties (Itoh et al., 1993; Dimitrova et al., 2008). While we did not address biologically plausible termination and branching conditions, we did use the contextual laminar architecture as a cue to set layer specific branching probabilities, and fronts in close proximity to the pia were terminated. Another way of restricting virtual morphologies is by generating them inside a limited space as applied here to the neurons generated in isolation. In such cases, a neurite terminates once it leaves the designated space (Cuntz et al., 2010; Memelli et al., 2013). This might explain in part why the neurons generated in isolation and in a limited space show less variance (**Figures 3H,I** and **4E,F**). However, since one of the future goals of this work is to generate full circuits, and because synapse occurrence is proportional to structural overlap between axons and dendrites (Peters and Feldman, 1976), we cannot constrain the space and generate large ensembles of neurons simultaneously (as in the forest setting, **Figures 3G** and **4C**). Therefore, future work will also focus on the design of proper rules for branching and termination.

We can also start an argument by assuming that the exemplar is not representative for all neurons. It has been demonstrated that reconstructed neurons contain a lot of biases related to reconstruction methods and selection by the experimenter (Horcholle-Bossavit et al., 2000; Kaspirzhny et al., 2002; Szilágyi and De Schutter, 2004; Steuber et al., 2004). For instance, the experimenter might select only “typical” neurons that are labeled well in the slice, which leads to a strong bias in the data. Also, neurons at the edge of a slice are more likely to be selected for technical reasons while precisely these neurons might be affected by the slice preparation in that neurites might be cut. Because these biases are not documented it is hard to make an estimate of their effect on the sample. As such, another option remains to explain the large variance in the generated data remains: the construction rules can be incomplete. Clearly the rules employed in this work are phenomenological and only crudely mimic morphological traits, so are incomplete. But assuming the rules are correct has interesting implications mainly because of the predictive power associated with a mechanistic model. Having a mechanistic explanation of neuron morphology has the advantage that morphological traits of various kinds can be predicted. For instance, age has an influence on morphologies and makes classifying neurons of varying age to correct classes nearly impossible (but see da Fontoura Costa et al., 2002). With a mechanistic model, morphologies corresponding to a certain age could be generated and serve as ground truth. Similarly, to assess pathological cases, simulated knock-outs could be predicted. Predictions, in turn, could be used to validate the phenomenological construction rules: predict the outcome of a particular knock-out and compare the resultant traits *in silico* and *in vitro.*

In conclusion, we designed, implemented and validated a new computational framework in accordance to a paradigm shift in the study of neuronal morphologies: away from studying morphologies in isolation to a study of neuronal morphologies as participants in their neuronal context. We demonstrated the potential of this new framework to study variation in neuronal morphology through a “generative” approach. Future research will focus on the generation and emergence of complete micro-circuits.

## Conflict of Interest Statement

The authors declare that the research was conducted in the absence of any commercial or financial relationships that could be construed as a potential conflict of interest.
